# Biodegradation of Dispersed Oil in Arctic Seawater at -1°C

**DOI:** 10.1371/journal.pone.0084297

**Published:** 2014-01-08

**Authors:** Kelly M. McFarlin, Roger C. Prince, Robert Perkins, Mary Beth Leigh

**Affiliations:** 1 Institute of Arctic Biology, University of Alaska Fairbanks, Fairbanks, Alaska, United States of America; 2 ExxonMobil Biomedical Sciences, Inc., Annandale, New Jersey, United States of America; 3 Institute of Northern Engineering, University of Alaska Fairbanks, Fairbanks, Alaska, United States of America; J. Craig Venter Institute, United States of America

## Abstract

As offshore oil and gas exploration expands in the Arctic, it is important to expand the scientific understanding of arctic ecology and environmental impact to mitigate operational risks. Understanding the fate of oil in arctic seawater is a key factor for consideration. Here we report the chemical loss due to the biodegradation of Alaska North Slope (ANS) crude oil that would occur in the water column following the successful dispersion of a surface oil slick. Primary biodegradation and mineralization were measured in mesocosms containing Arctic seawater collected from the Chukchi Sea, Alaska, incubated at −1°C. Indigenous microorganisms degraded both fresh and weathered oil, in both the presence and absence of Corexit 9500, with oil losses ranging from 46−61% and up to 11% mineralization over 60 days. When tested alone, 14% of 50 ppm Corexit 9500 was mineralized within 60 days. Our study reveals that microorganisms indigenous to Arctic seawater are capable of performing extensive biodegradation of chemically and physically dispersed oil at an environmentally relevant temperature (−1°C) without any additional nutrients.

## Introduction

As the oil and gas industry continues offshore exploration in the Arctic, it is imperative to base design and operational plans on a deep scientific understanding of the arctic ecology and the potential environmental impact in order to mitigate risks. The Arctic’s fragile environment and sensitive ecology present unique challenges. Understanding the rate and extent of oil biodegradation in cold water environments is a key factor for consideration. Biodegradation is generally believed to be the dominant process that removes petroleum compounds from the environment [1], but the process has not been thoroughly studied in the Arctic, and questions remain as to whether biodegradation is a significant process in cold conditions [2]. Microorganisms capable of using hydrocarbons as a source of carbon and energy are diverse and widespread [3], including in the Arctic [4] and other cold environments [5]–[8].

Dispersant application is a potential oil spill response option in Arctic marine environments. Corexit 9500 has been shown to be effective in dispersing Alaska North Slope (ANS) crude oil in a large outdoor wave tank at temperatures ranging from 0−3°C [9]. When Corexit 9500 was widely applied to the Deepwater Horizon spill, some research suggested that the dispersant was not toxic to the indigenous Gulf of Mexico microorganisms and that some bacterial species were capable of degrading various components of the dispersant [10]. However, little is known about the degradation of Corexit 9500 and dispersed oil in the Arctic marine environment.

When a dispersant is applied to an oil slick it reduces the interfacial surface tension between the water and the oil, allowing the oil to become mixed into the water column as tiny (1−70 µm) droplets [11] with mild wave action. The creation of small oil droplets increases the surface area available for microbial colonization [12] and can significantly increase biodegradation [11], . The localized concentration of oil drastically decreases as it is chemically dispersed into the water column and has been reported to range from 1−15 ppm beneath (1−5 m) oil slicks treated with dispersant within a few hours after the application [13], . This study aimed to assess the biodegradation of oil at concentrations that are expected in the water column following successful dispersion in the Chukchi Sea.

Several laboratory studies have investigated sub-Arctic oil biodegradation in Alaska [20], [21], Canada [5], and Arctic and sub-Arctic Norway [6], [8], [22], but with diverse experimental methodologies, making direct comparisons of biodegradation data challenging. Previous studies have used a range of oil loadings and in some cases the addition of enrichment cultures and/or high quantities of nutrient amendments. Most experiments have been conducted using high dispersed oil concentrations (100−900 ppm) [19], [21], [23], while few studies have focused on the low dispersed oil concentrations (1−15 ppm) that soon occur beneath a dispersed slick [13], . Other studies have supplemented oil incubations with large quantities of nutrients, which accelerate biodegradation rates [24]–[26] but may create an experimental system that does not accurately represent environmental conditions.

This study measures the biodegradation of Alaska North Slope crude oil [27] that would occur in the water column following a successful application of Corexit 9500 to a surface oil slick. Arctic seawater with its indigenous microbial community was collected and experiments were conducted at the temperature of the water at the time of collection (−1°C) with natural or slightly enhanced nutrient levels. Biodegradation was measured using respirometry and gas chromatography-mass spectrometry (GC-MS) analysis.

## Methods

Field studies did not involve endangered or protected species. Water collection did not require specific permission because the Chukchi Sea is not a federal or state protected area.

### Seawater Collection

Seawater (free of slush and ice) was collected from the eastern edge of the Chukchi Sea, approximately 2.5 km East of where the Chukchi Sea meets the Beaufort Sea. Samples were collected 1 km from Barrow, AK (N 71°21’43”, W 156°40’13”) from beneath 1 m of ice. Seawater was immediately transferred to the laboratory cold-room in clean Nalgene® rectangular carboys and aerated with aquarium air stones until test initiation. The test waters did not contain visible particulates and were not filtered. Measured seawater quality parameters at collection included pH (8.05), temperature (−1°C), dissolved oxygen (11.6 mg/L) and salinity (33 ppt). Nutrient levels (nitrate, nitrite, and ammonia) were below detection limits by simple colorimetric tests (DR/850, Hach, Colorado).

### Experimental Environmental Parameters

All incubations were performed in a cold room, which was kept at −1°C, and under low light (Photosynthetically Active Radiation (PAR) of 1.83 µmol·s^−1^·m^−2^; LI-193SA Spherical Quantum Sensor, LI-COR, Nebraska).

### Oil and Dispersant

A single batch of Alaska North Slope (ANS) crude oil was collected from the Alyeska terminal in 2009. Some was artificially weathered by allowing a known weight of static fresh oil to evaporate (at room temperature) in a fume hood until it had lost 20% of its initial mass to approximate oil that might have been floating at sea for 12−24 hours [28]. The dispersant used in this study was Corexit 9500 [29]. Because of the small volumes involved, oil and dispersant were premixed before addition to the experimental chambers. The biodegradation of dispersed oil was tested at 1∶20 and 1∶15 dispersant to oil ratios (DORs) and the mineralization of Corexit 9500 alone was measured at 50 ppm. The 1:20 DOR application rate is the target ratio in oil spill response, although ratios as high as 1∶10 have been required with more emulsified and viscous heavy oils [19].

### Biodegradation experiments

Low concentrations of oil were tested (2.5 ppm and 15 ppm) in order to assess the biodegradation of dispersed oil at concentrations that are expected to approach those found in the water column after successful dispersion. Two methods were used to quantify the biodegradation of oil. The first measured the primary biodegradation of the oil, i.e. the chemical disappearance of specific hydrocarbons, monitored with respect to a conserved internal marker within the oil (hopane) [30]. Primary biodegradation was measured in open top mesocosms (2.5 ppm oil in 4 L of unamended seawater) and closed respirometer flasks (15 ppm oil in 900 ml seawater + nutrients). Respirometer flasks contained their own oxygen generation system. The second method measured the mineralization of oil to CO_2_ and H_2_O with an electrolytic respirometer (Co-ordinated Environmental Service; Kent, England) and required the use of higher oil concentrations (15 ppm oil in 900 ml seawater + nutrients) because of the detection limits of the instrument. Preliminary experiments indicated that the biodegradation of such concentrations (15 ppm) would likely be limited by the availability of nitrogen and phosphorus in the seawater, so low levels of nutrients (16 ppm Bushnell Haas Broth [31]) were added to respirometer flasks, providing 75 µM phosphate, 49 µM nitrate and 76 µM ammonium. For comparison, Codispoti *et al.* [32] measured background nutrient levels adjacent to Point Barrow and reported 0.8 µM phosphate, 6 µM nitrate and 6 µM ammonium in summer surface seawater.

All treatments were continuously mixed (400 rpm) using Teflon coated stir-bars. Respirometer experiments were carried out in general agreement with the Organization for Economic Cooperation and Development 301F [33] guidelines for biodegradation testing, with the omission of a microbial inoculum. Mineralization was determined as a function of the sample’s theoretical oxygen demand (ThOD): the amount of O_2_ required to mineralize the sample. ThOD was calculated based upon analytical measurements of the substrate’s elemental composition (QTI, Whitehouse, NJ): ANS crude oil contained 84.9% carbon, 12.0% hydrogen, and 0.39% nitrogen (ThOD  =  3.32 mg O_2_/mg oil), while oil plus Corexit 9500 (20:1) contained 83.8% carbon, 11.6% hydrogen, and 0.32% nitrogen (ThOD  =  3.30 mg O_2_/mg dispersed oil). Positive controls for the respirometry contained either sodium benzoate or peptone at 50 ppm. Negative controls for the respirometry experiments contained seawater and 0.5% BH with no oil or dispersant addition. Materials necessary for sterile controls were unavailable in our remote Arctic research facility. The minimal respiration measured in the negative controls (3 replicates) was subtracted from all respirometer treatments.

### Oil Analyses

Petroleum hydrocarbons were extracted from all experimental incubations and analyzed with GC-MS as described by Douglas *et al*. [34]. Oil biodegradation was determined with respect to 17α(H),21β(H)-hopane as a conserved internal marker within the oil [30]. Each experimental container was extracted three times with methylene chloride. The combined extract was concentrated to a nominal concentration of approximately 10 mg/ml by evaporation to a small volume (but not to dryness), then dried of water and filtered by passage through a column of anhydrous sodium sulfate. All treatments were analyzed for total detectable hydrocarbons as well as individual aromatics and alkanes.

### Statistical analyses

Levene's test for equality of variances and the t-test for equality of means were performed with a 95% confidence interval using a statistical package for social science, version 16 (SPSS Inc., Chicago, IL, USA).

## Results and Discussion

**Figure 1:**
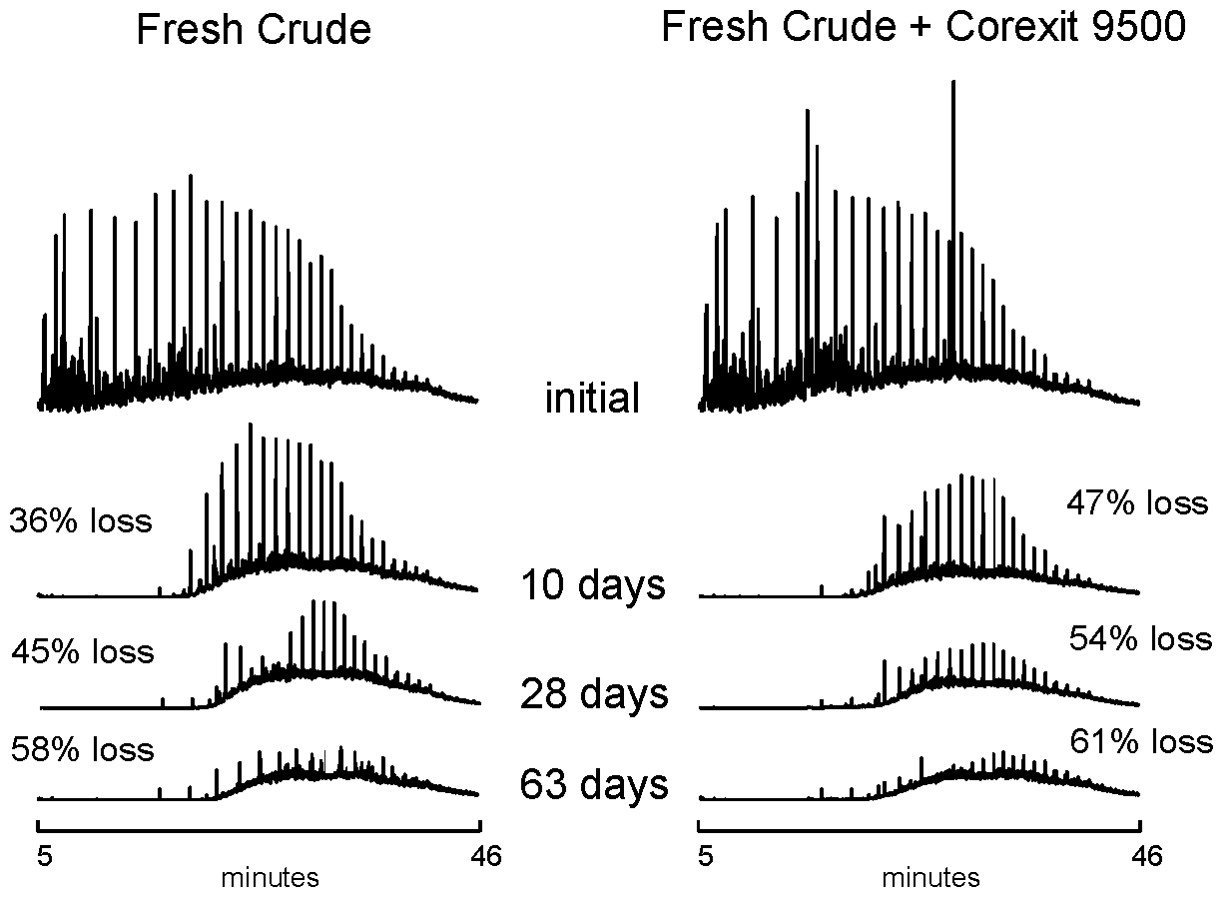
GC-MS total ion chromatograms. Open top mesocosm incubations contained an initial loading of 2.5 ppm ANS crude oil without and with Corexit 9500 (1∶15 DOR), and no added nutrients. Chromatograms show the pattern of biodegradation after 10 days, 28 days and 63 days.

**Figure 2:**
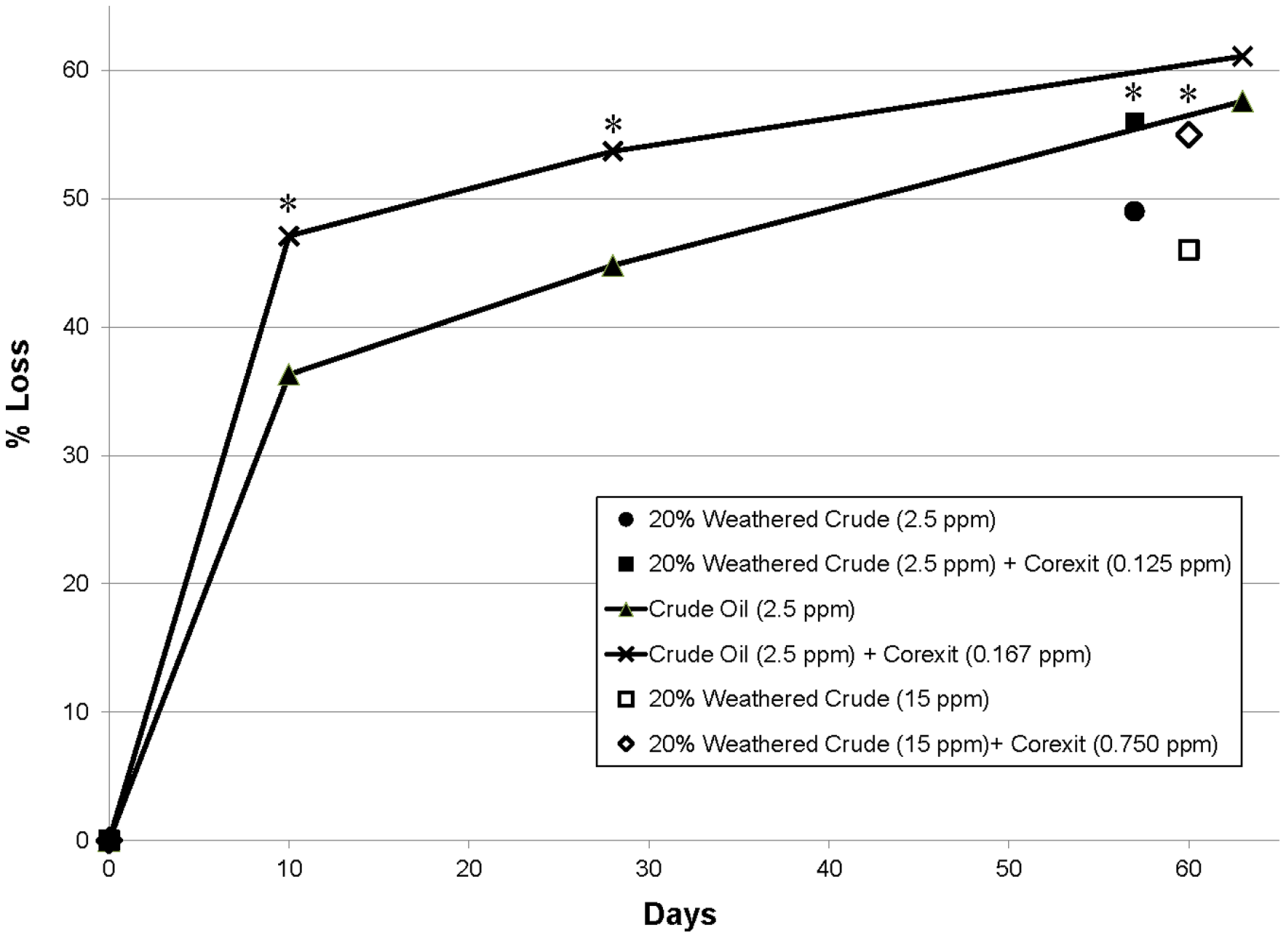
Primary biodegradation as measured by loss of total measurable petroleum hydrocarbons at −1°C. The higher concentrated (15 ppm) weathered oil treatments contained a small amount of nutrient supplementation (16 ppm Bushnell Haas), and were incubated in a sealed flask for parallel respirometry measurements (Fig 4). All other treatments had no added nutrients and were open to the atmosphere. Results are normalized to hopane. Standard deviations are reported in Table 1. *Denotes a significant difference between treatments with and without Corexit 9500 for each test (p<0.05).

The primary goal of this study was to quantify the chemical loss due to the biodegradation of dispersed oil at low temperature by indigenous microorganisms in Arctic seawater. Extensive biodegradation of ANS crude oil occurred at −1°C, both in the presence and absence of the dispersant Corexit 9500 (Figure 1). By the end of the incubation period (∼60 days), 46−61% of total measurable oil was lost (Figure 2, Table 1). Percent losses were affected by prior weathering of oil and by the presence of Corexit 9500.

**Table 1:** Mineralization and primary biodegradation of Corexit 9500 and ANS crude oil as determined by respirometry and GC-MS analysis.

Test ID	Treatments	Bushnell Haas (mg/L)	Dispersant Concentration (mg/L)	Oil Concentration (mg/L)	% Loss of Detectable Hydrocarbons	% Mineralized
**Respirometer Flasks (900 ml seawater)**						
R2	Corexit Only	0.016	50.0	na	nm	14
R1	20% Weathered	0.016	na	15	46±5.1	11
	20% Weathered + Corexit (1∶20 DOR)	0.016	0.750	15	55±1.5*	11
**Mesocosms (4 L seawater)**						
M2	Fresh Oil	na	na	2.5	58±10	nm
	Fresh Oil + Corexit (1:15 DOR)	na	0.167	2.5	61±0.45	nm
M1	20% Weathered	na	na	2.5	48±1.5	nm
	20% Weathered + Corexit (1:20 DOR)	na	0.125	2.5	57±4.2*	nm

All treatments were incubated at −1°C.

Denotes significant difference (p<0.05) between treatments in each test, na: not applicable, nm: not measured. Standard deviations are presented for primary biodegradation.

**Figure 3:**
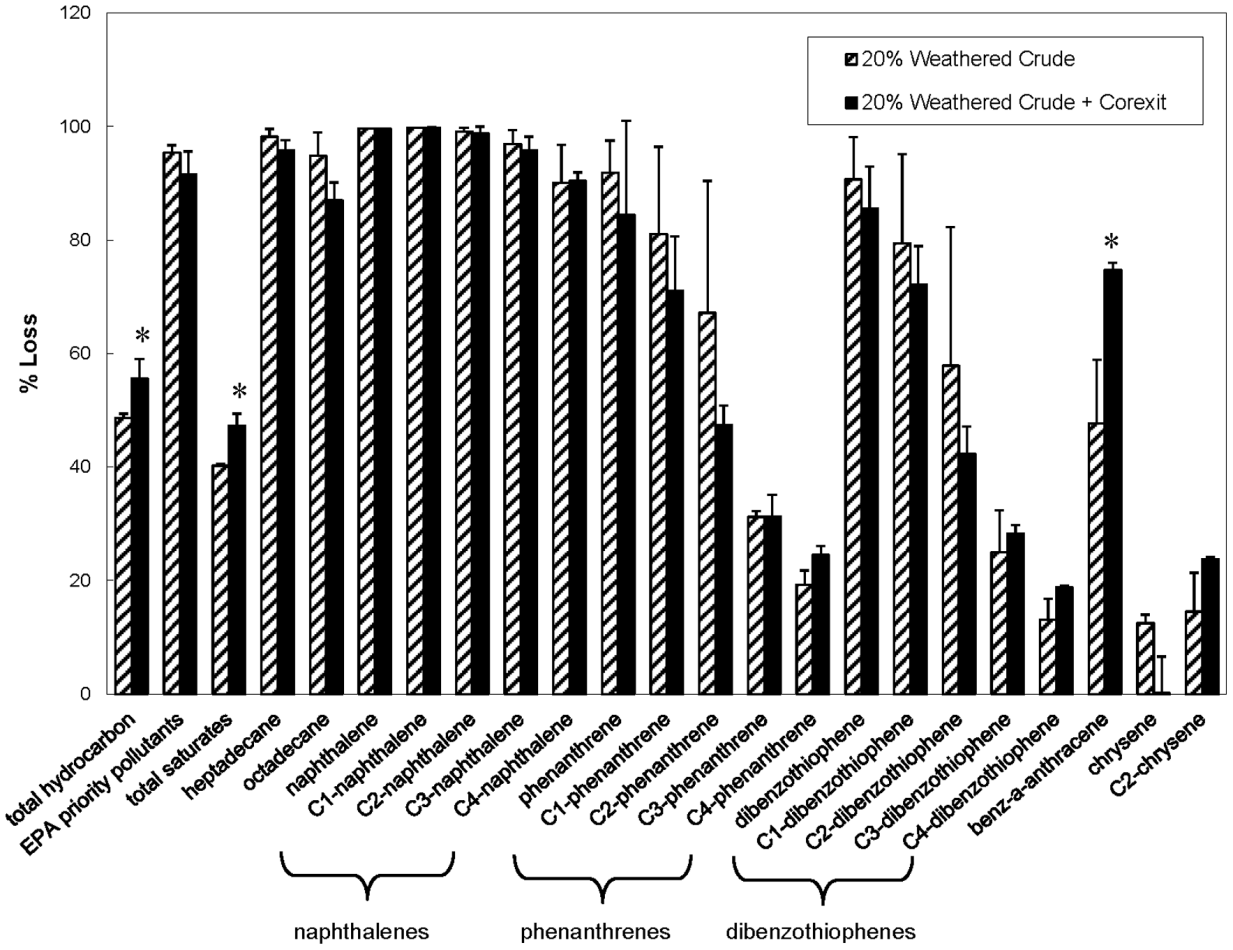
Percent loss of weathered oil after 56 days without nutrient addition at −1°C. Open top mesocosms contained an initial concentration of 2.5 ppm of 20% weathered oil with and without the chemical dispersant Corexit 9500 at a 1∶20 DOR. Standard errors are displayed. Results are normalized to hopane. *Denotes a significant difference between treatments with and without Corexit 9500 (p<0.05).

**Figure 4:**
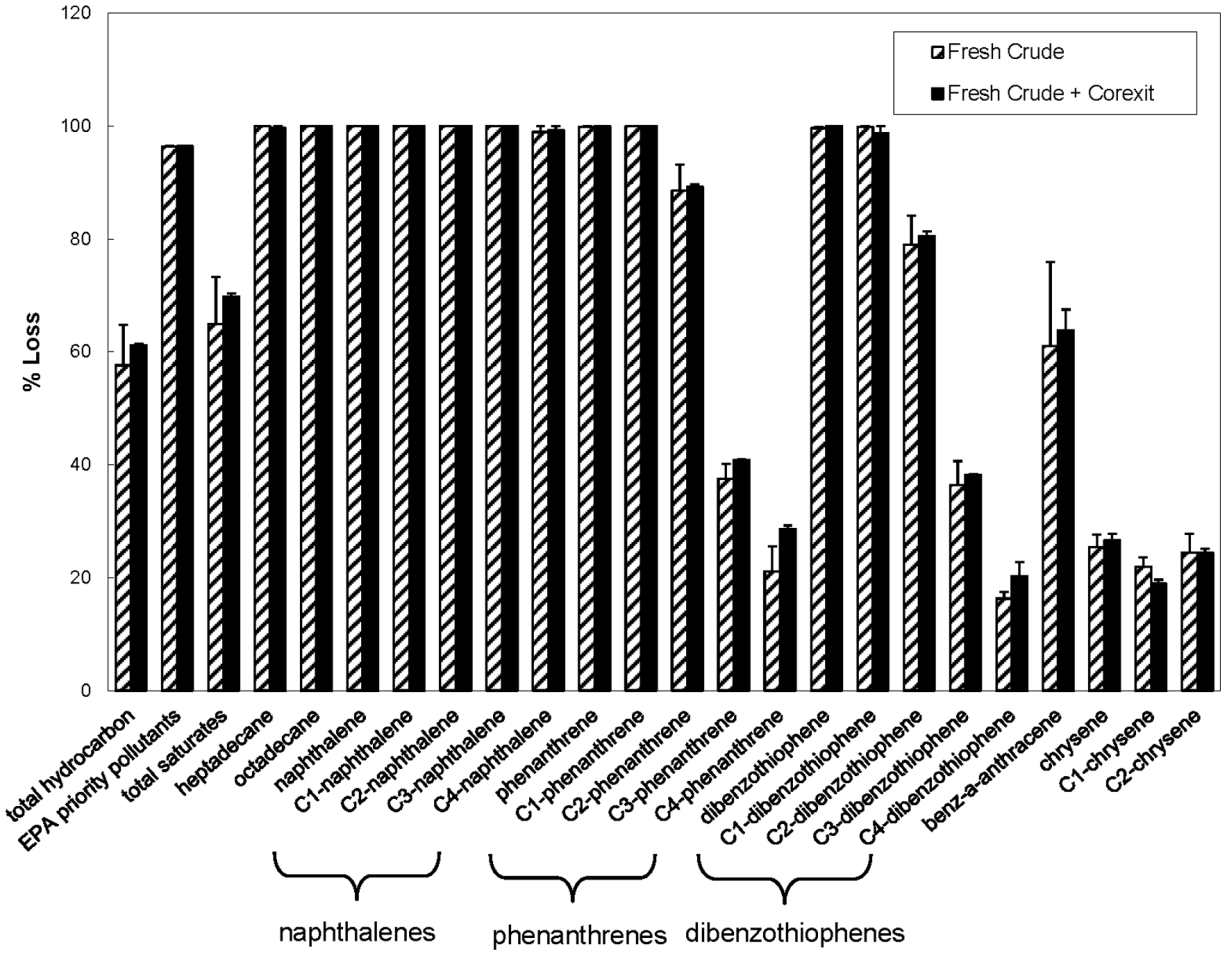
Percent loss of fresh oil after 63 days without nutrient addition at −1°C. Open top mesocosms contained an initial concentration of 2.5 ppm of fresh oil with and without the chemical dispersant Corexit 9500 at a 1:15 DOR. Standard errors are displayed. Results are normalized to hopane. No significant differences (p<0.05) were observed.

As reported by Venosa and Holder [21] at warmer temperatures, Corexit 9500 initially enhanced the rate of biodegradation, although differences between treatments with and without Corexit became smaller as time progressed (Figure 2). At the end of the experiments (56−63 days), the indigenous Arctic microbial community (in incubations containing 2.5 ppm fresh and weathered oil), had biodegraded almost 100% of heptadecane, octadecane, and individual aromatics; including EPA listed priority pollutants (Figure 3 & 4) regardless of the presence of Corexit 9500. In addition, the complete loss of phenanthrene, C-1 phenanthrenes, dibenzothiophenes and C1-dibenzothiophenes was observed with or without Corexit in fresh oil incubations (Figure 4). The loss of C2-phenanthrenes was close to 90%, while almost 80% of C2-dibenzothiophenes was removed, as was approximately 60% of benz[a]anthracene (Figure 4). In addition, the indigenous microbial community was able to biodegrade the four-ringed PAH, chrysene (Figure 4). As expected, the degradation rates were lower for chrysene than for the lower molecular weight compounds (e.g., naphthalene, phenanthrene; Figures 3 & 4). The patterns of petroleum hydrocarbon biodegradation observed in these experiments were similar to those observed previously in both sub-arctic and temperate conditions [34]; with the shorter, straight-chained alkanes more readily degraded than longer *n*-alkanes and branched alkanes, lower molecular weight PAHs more readily degraded than higher molecular weight PAHs, and parent PAHs degraded before their alkylated homologues.

It is not surprising that at such low oil concentrations only small differences in biodegradation were observed with the addition of Corexit 9500, although some have expressed concern that Corexit might be inhibitory [20], [35]. The desire to test equivalent oil loadings for both dispersant treated and untreated mesocosms resulted in both treatments having very similar quantities of dispersed oil, as the small volumes of oil added to the mesocosms dispersed naturally without Corexit addition due to the physical mixing. In the environment, it is unlikely that a large oil slick would be physically dispersed to these low concentrations without dispersant application. The important finding of this work is that indigenous Arctic marine microorganisms are capable of extensive biodegradation of dispersed oil, regardless of whether physically or chemically dispersed. We note, though, that dispersants such as Corexit 9500 dramatically increase the amount of dispersed oil in the water column, and minimize surface slicks.

The oil losses detected in this study are primarily due to biodegradation, although volatilization may be responsible for a portion of the loss of low molecular weight compounds in fresh oil. The chromatograms shown in Figure 1 are normalized to an internal marker within the oil (17α(H),21β(H)-hopane) [30], so the losses represent the primary biodegradation of total extractable hydrocarbons rather than, for example, physical loss due to poor extraction. In addition to normalizing GC-MS data to conservative markers, such as hopane, reporting ratios of degradable hydrocarbons (alkanes or PAHs) to recalcitrant hydrocarbons (isoprenoids) has also been used as a measure of biodegradation [15], [36], [37]. Table 2 summarizes the C_18_ to phytane ratio at 10, 28 and 63 days in mesocosms containing 2.5 ppm of oil with and without Corexit 9500. The ratio decreases with time, as expected during biodegradation.

**Table 2:** Biodegradation index (C_18_/Phytane) of mesocosms containing 2.5 ppm fresh ANS crude oil without nutrient addition incubated at −1° with and without Corexit 9500 (DOR 1:15) analyzed at 10, 28 and 63 days.

	C18/Phytane	
	Crude Oil	Crude Oil + Corexit 9500
**Initial Oil**	1.8±0.03	1.8±0.03
**10-day**	1.7±0.04	0.97±0.13
**28-day**	0.098±0.1	0.082±0.02
**63-day**	0.009±0.004	0.003±0.0001

Biodegradation of dilute oil is extensive in temperate [38], [39], tropical [40] and Arctic environments (this study), although the rates of biodegradation vary based on region. By the end of our incubations (∼60 days), 46−61% of total GC-detectable oil had been lost (Figure 2). Thus the overall ‘half-life’ of crude oil in the Chukchi Sea at −1°C, when present at low concentrations (2.5−15 ppm), was on the order of 60 days. Using the same methods as our study, Prince *et al*. [39] measured an 82−88% loss of weathered ANS crude oil without and with Corexit 9500 in incubations with New Jersey seawater at 8°C after 60 days. They also measured the % loss of fresh oil and concluded that 81−82% of oil was lost after 41 days, which corresponds to an 11−14 day half-life with and without Corexit, respectively. Zahed *et al.* [40] also measured the half-life and percent loss of crude oil (100 mg/L) in incubations with indigenous seawater enrichment cultures from Butterworth, Malaysia with and without Corexit 9500 at 28°C. In tropical seawater and with a light crude oil, they reported a maximum loss of 67 and 64% of total petroleum hydrocarbons over 45 day incubations, with half-lives of 28 and 31 days, respectively. Hazen *et al*. [38] reported half-lives of soluble alkanes in MC252 oil dispersed with Corexit 9500 in the Deepwater Horizon subsurface (1099−1219 m) plume to range from 1.2−6.1 days at a temperature of 5°C. Of course the half-life of petroleum does not predict the persistence of total oil, as the low molecular weight hydrocarbons are more readily biodegraded than the larger compounds. Half-life measurements are dependent on many factors, with oil concentration and composition being very important, making direct comparisons among different studies using different oil loadings and types challenging. However, all these studies show that indigenous microbial communities are able to degrade a substantial amount of petroleum in tropical, temperate and Arctic environments.

Crude oils are very dense sources of carbon and energy that provide no biologically available nitrogen or phosphorus, which are essential for microbial growth. If oil is stranded on shorelines, this limitation can be overcome by the application of fertilizers [41], even in the Arctic [42]. When successfully applied to an oil slick, dispersants dilute the oil so that the natural background levels of nutrients in the sea may support the microbial community during oil biodegradation [39]. In our 60-day experiments, 4 L mesocosms containing 2.5 ppm oil and no added nutrients exhibited similar oil biodegradation extents as smaller (900 ml) respirometer flasks containing 15 ppm oil and additional nutrients (16 ppm Bushnell Haas medium). Because the aim of the study was to replicate environmental conditions as much as possible, higher concentrations of nutrients were not tested to evaluate whether additional nutrients might accelerate biodegradation.

**Figure 5:**
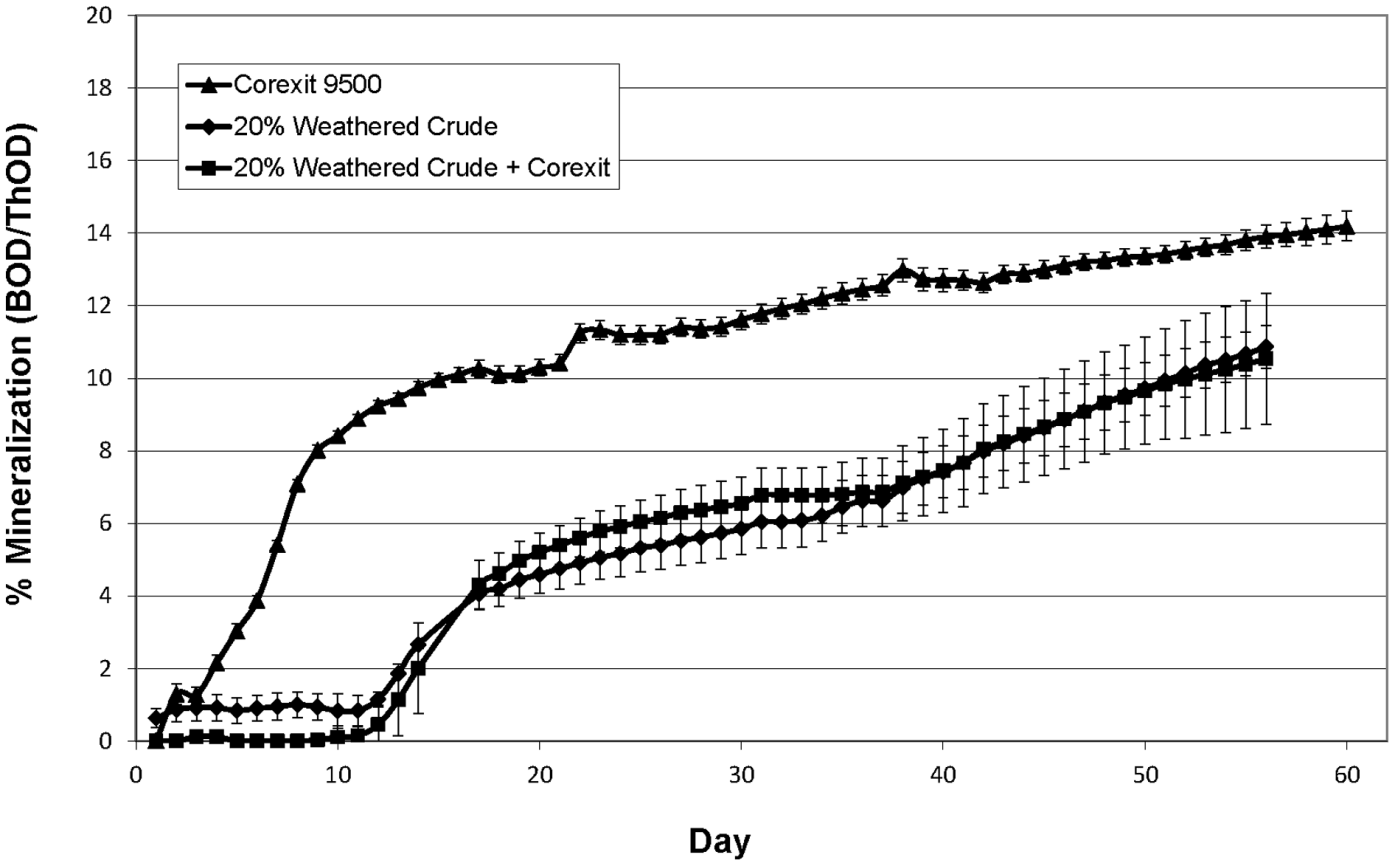
Percent mineralization at −1°C. Sealed respirometer experiments contained Corexit 9500 (50 ppm) alone or 20% weathered ANS crude (15 ppm) with and without Corexit 9500 (1∶20 DOR). All treatments contained seawater and 16 ppm of Bushnell Haas.

In addition to measuring primary biodegradation (loss of individual chemicals) using GC/MS, we also measured mineralization (i.e. the complete respiration of substrate to CO_2_ and H_2_O) with respirometry. Although primary biodegradation was slightly enhanced by Corexit 9500 (Figure 2), no stimulatory effect of the dispersant was observed for mineralization (Figure 5). The indigenous microbial community mineralized 11% of weathered crude oil (15 ppm) after 56 days in both physically and chemically dispersed treatments. Lindstrom and Braddock [20] compared the mineralization of oil with and without dispersant using a microbial enrichment culture from a sub-Arctic spill site, and similarly concluded that Corexit 9500 had little effect on mineralization. Baelum *et al*. [15] also saw no significant difference in mineralization rates in incubations containing MC252 oil with and without Corexit 9500 at 5°C after 20 days, but reported a significant increase in primary biodegradation (60% vs. 25%) with the addition of Corexit.

There are several factors contributing to the fact that percent mineralization will likely always be lower than primary degradation. One is that oil-degrading bacteria utilize petroleum as a carbon source, integrating a portion of hydrocarbon metabolites directly into biomass [43]–[45]. Another is that GC analyses only measure a fraction of the hydrocarbons in most oils, while % mineralization is based on total oil. The resin and polar fractions of the oil [46] are not significantly volatile, and do not enter the GC column, and nor do hydrocarbons with more than about 40 carbon atoms. All of these molecules are expected to degrade more slowly than hydrocarbons with <30 carbon atoms, although evidence is accumulating that resins and asphaltenes are at least partially degradable [47], [48]. Taking these two effects into consideration, our respirometry data are not inconsistent with our GC data.

Biodegradation of the dispersant alone was also examined using respirometry. Primary biodegradation of Corexit 9500 was not assessed, since solvent extraction and the GC/MS methods used in this study do not accurately measure all the components present in the dispersant. The concentration of Corexit 9500 (50 ppm) in dispersant-only incubations was considerably higher than the concentration of Corexit in chemically dispersed oil incubations (0.75 ppm) to enable detection of mineralization, and mineralization may have been nutrient limited due to the use of such a high concentration in our low nutrient incubations. Nevertheless, approximately 14% of Corexit 9500 (50 ppm) was mineralized by the indigenous Arctic marine microbial community within 60 days at −1°C (Figure 5). Incubations with Corexit 9500 alone (50 ppm) consumed more oxygen and at a much faster rate than treatments containing oil (15 ppm oil + 0.75 ppm Corexit). The rate of oxygen consumption in the Corexit 9500 treatment (50 ppm) was the greatest over the first 10 days, while the treatments containing 15 ppm weathered ANS and weathered ANS plus Corexit (15 ppm oil + 0.75 ppm Corexit) reached a maximum rate of oxygen consumption between day 12 and day 16, respectively. Almost no lag period was observed for the mineralization of Corexit 9500 alone (Figure 5), suggesting that the indigenous microbial community can readily initiate biodegradation of at least some components of the dispersant. The overall mineralization pattern of ANS and Corexit observed in these experiments are similar to the results of Lindstrom and Braddock [20], who reported that Corexit 9500 was mineralized faster than fresh ANS crude, which in turn was mineralized faster than weathered ANS crude. Future studies using analytical methods capable of measuring chemical losses of dispersant components, such as LC-MS, would enable a more thorough understanding of Corexit 9500 biodegradation.

## Conclusion

To our knowledge, this is the first study to measure the biodegradation of a crude oil, with and without a dispersant, at environmentally relevant concentrations [17] by an indigenous Arctic microbial community at sub-zero temperatures. Microorganisms indigenous to the Chukchi Sea were found to degrade both fresh and weathered crude oil in the presence and absence of Corexit 9500 at −1°C, with oil losses ranging from 46−61% and up to 11% mineralization over 60 days. Weathered ANS dispersed with Corexit 9500 underwent a 57% loss in Arctic seawater after 60 days in our experiment, but experienced an 88% loss in New Jersey seawater in the same time [39]. These experiments suggest that in the Arctic, ANS crude oil degrades more slowly than oil in temperate regions, but that oil losses were still substantial even at −1°C. There is evidence that Corexit 9500 initially stimulated oil biodegradation (Figures 1 and 2), but, as expected, its effects were minimal in longer term incubations. We conclude that the biodegradation of oil in Arctic seawater is extensive at −1°C, and that the biodegradation of dilute, dispersed oil is not inhibited by the presence of Corexit 9500. Although no microbial analyses are reported, it is apparent that the chemical loss of oil is indeed microbial. The respiration measured in all treatments could only be the result of indigenous microorganisms mineralizing oil and/or dispersant, since the minimal respiration measured in the background controls (seawater + nutrients) was subtracted from the respiration measured in the treatments (seawater + nutrients + oil and/or dispersant). Furthermore, the selective disappearance of some chemicals before others, whether referred to hopane, or for example the older C_18_ to phytane ratio, is a diagnostic for biodegradation [49]. Future work will focus on biodegradation rates in offshore Arctic oil lease areas and on the identification of microorganisms and genes active in biodegradation. Additional research in the Arctic is needed to address the behavior and biodegradation of oil spilled in ice covered waters.
